# Fetal Exposure to PCBs and Their Hydroxylated Metabolites in a Dutch Cohort

**DOI:** 10.1289/ehp.6424

**Published:** 2004-04-13

**Authors:** Shalini Devi Soechitram, Maria Athanasiadou, Lotta Hovander, Åke Bergman, Pieter Jan Jacob Sauer

**Affiliations:** ^1^University Hospital Groningen, Department of Paediatrics/Beatrix Children’s Hospital, Groningen, the Netherlands; ^2^Department of Environmental Chemistry, Stockholm University, Stockholm, Sweden

**Keywords:** cord blood, human plasma, hydroxylated PCBs, PCB, placental transfer

## Abstract

Polychlorinated biphenyls (PCBs) are still the most abundant pollutants in wildlife and humans. Hydroxylated PCB metabolites (OH-PCBs) are known to be formed in humans and wildlife. Studies in animals show that these metabolites cause endocrine-related toxicity. The health effects in humans have not yet been evaluated, especially the effect on the fetus and newborn. The aim of this study is to measure the levels of PCBs and OH-PCBs in maternal and cord blood samples in a population with background levels of PCBs. We analyzed 51 maternal and corresponding cord blood samples in the northern part of the Netherlands. The PCB concentrations in maternal plasma ranged from 2 to 293 ng/g lipid, and OH-PCB concentrations from nondetectable (ND) to 0.62 ng/g fresh weight. In cord plasma, PCB concentrations were 1–277 ng/g lipid, and OH-PCB concentrations, ND to 0.47 ng/g fresh weight. The cord versus maternal blood calculated ratio was 1.28 ± 0.56 for PCBs and 2.11 ± 1.33 for OH-PCBs, expressed per gram of lipid. When expressed per gram fresh weight, the ratios are 0.32 ± 0.15 and 0.53 ± 0.23 for PCBs and OH-PCBs, respectively. A significant correlation between the respective maternal and cord levels for both PCBs and OH-PCBs was found. Our results indicate that OH-PCBs and PCBs are transferred across the placenta to the fetus in concentrations resulting in levels of approximately 50 and 30%, respectively, of those in maternal plasma. More research in humans is needed to evaluate potential negative effects of these endocrine disruptors on the fetus.

Polychlorinated biphenyls (PCBs) are, together with DDT (dichlorodiphenyltrichloroethane) and DDT-related chemicals, the most dominating classes of environmental pollutants worldwide, with concentrations varying in wildlife and in humans from different areas of the globe (Damstra et al. 2002). Although all PCB congeners, either present in commercial mixtures or as single chemicals, are lipophilic substances with low water solubility, only some of them, even within the same class of chemicals, have a strong tendency to accumulate in higher organisms. This is shown by a few strongly dominating PCB congeners retained in, for example, humans (Fängström et al. 2002; Koopman-Esseboom et al. 1994a), grey seals (Blomkvist et al. 1992), and polar bears (Letcher et al. 1996), all acting at top of the food chain. PCB concentrations are generally in the low micrograms PCB per gram lipid range among humans (Koopman-Esseboom et al. 1994b; Sjödin et al. 2000), but higher levels are occasionally found in individuals with a heavy consumption of fatty fish from contaminated waters (Sjödin et al. 2000) or with a pronounced diet based on subsistence food items (Ayotte et al 1997; Fängström et al. 2002). The levels of PCBs have been shown to slowly decrease (Norén and Meironyté 2000) as a result of the legislative measures taken to prohibit the production of PCBs in the early 1970s, leading to lower environmental releases.

Studies have shown negative effects of PCBs in animals and humans, especially in newborn infants (Patandin 1999; Winneke et al. 2002). Reported effects of background exposure in infants include reduced birth weight, less postnatal growth (Patandin et al. 1998; Rylander et al. 1996), neonatal hypotonia (Huisman et al. 1995a, 1995b), impaired development and impaired immune response (Weisglas-Kuperus 1998; Weisglas-Kuperus et al. 1995), and lower thyroid hormone levels (Brouwer et al. 1998, 1999; Koopman-Esseboom et al. 1994a, 1997; Osius et al. 1999). With few exceptions (e.g., Walkowiak et al. 2001), most negative effects of background levels of PCBs were primarily related to antenatal exposure, whereas post-natal effects from PCBs were related mainly to accidental exposure of infants to rather high levels of PCBs and other organohalogen substances (Kuratsune et al. 1996). It is unknown whether these effects are caused by the PCBs themselves or by their metabolites.

Hydroxylated PCBs (OH-PCBs), or polychlorobiphenylols, are major metabolites of PCBs (Letcher et al. 2000). These metabolites are formed by oxidative metabolism of PCBs, mediated by the cytochrome P450 enzymatic system, that generally involves an arene oxide intermediate (Jerina and Daly 1974). OH-PCBs, like most phenolic compounds, are readily conjugated and excreted, but several OH-PCB congeners and some other halogenated phenolic compounds have been found to be retained in human and wildlife blood (Bergman et al. 1994; Fängström et al. 2002; Hovander et al. 2002; Letcher et al. 2000; Sandau et al. 2000). The OH-PCB concentrations so far reported have been 10–20% of the PCB level in humans but were found to be higher in, for example, polar bear blood (Sandau et al. 2000). The three most abundant OH-PCB congeners retained in the blood are metabolites of CB105, CB118, CB138, CB153, and CB187 (Hovander et al. 2002), all known to be among the most persistent and bioaccumulative PCB congeners.

The toxicologic impact of OH-PCBs is still not known, but several studies indicate that these metabolites may have adverse effects in mammals (Meerts et al. 2002). In animals, OH-PCBs are transferred across the placenta (Bjerregaard and Hansen 2000; Korrick et al. 2000; Sala et al. 2001). It is not yet known at what rate OH-PCBs are transferred to the human fetus (Meironyté-Guvenius et al. 2003). The objective of the present study was to assess PCB and OH-PCB levels in mothers and children at birth and to determine the transplacental transfer of PCBs and OH-PCBs.

## Materials and Methods

### Cohort.

From September 1998 through December 2000, pregnant women from the northern part of the Netherlands were invited by their midwife or obstetrician to participate in a study on exposure to PCBs and OH-PCBs and their potential effects on the development of the newborn infant. The mothers had to be of Western European origin, and Dutch had to be their native language. To establish an optimal study population, pregnancy and delivery had to involve no serious illness or complications. Only infants born at term (37–42 weeks of gestation) without congenital anomalies or diseases were included. Admission of an infant at a hospital more than 1 day after birth was an exclusion criterion. The medical ethics committee of the University of Groningen approved the study. A blood sample was taken from the pregnant women in the second and/or third trimester of their pregnancy. Blood samples of the umbilical cord were taken directly after delivery. Blood was collected in a vacuum system EDTA tube (Ritmeester, Utrecht, the Netherlands) and centrifuged within 24 hr for 5 min at 4,000 rpm. The plasma was transferred to separate glass tubes with screw caps with Teflon inlayers and stored at −18°C to −20°C until analysis. A total of 51 paired maternal and cord blood plasma samples were analyzed in the present study. An additional 29 maternal blood samples and 11 cord blood samples were also analyzed, but these samples were not paired and are not included in this study. The 51 samples were analyzed at the analytical laboratory participating in the study.

### Chemicals.

Hexane and dichloromethane were of pesticide grade (Fisons, Leicestershire, England). Methyl *tert*-butyl ether (MTBE), 2-propanol, and potassium hydroxide (Eka Nobel AB, Bohus, Sweden), as well as potassium chloride (Merck, Darmstadt, Germany) and sulfuric acid (98%; BDH Laboratory Supplies, Poole, England), were of analytic quality. Ethanol (99.5%) was purchased from Kemetyl (Haninge, Sweden). Diazomethane was synthesized as described by Furniss et al. (1989). Silica gel (< 0.063 mm; Macherey-Nagel, Düren, Germany) was activated by heating it overnight at 280°C and allowed to cool to room temperature before use. All glassware was heated at 300°C overnight before use.

### Instruments.

Gas chromatography (GC) was performed on a Varian 3400 GC equipped with an electron capture detector, a Varian 8200 autosampler, and a split/splitless injector operated in the splitless mode. The fused silica capillary column used was a nonpolar column, CP-SIL 8CB (25 m × 0.15 mm × 0.12 μm), from Chrompack (EA Middelburg, the Netherlands). The column oven temperature was programmed as follows: for analysis of methylated derivatives of OH-PCBs, 80°C (2 min), then 50°C/min to 200°C, then 1°C/min to 230C°, then 30°C/min to 330°C (3 min); for analysis of PCBs, 80°C (1 min), then 20°C/min to 300°C (10 min). The injector and detector temperatures were 250°C and 360°C, respectively. Hydrogen was used as carrier gas, and nitrogen was used as makeup gas.

For the evaporation of solvents during cleanup of the samples, we used a centrifugal concentrator (Genevac SF50 Sales Development Ltd., Ipswich, England). For the phase separation during the extraction and lipid removal with concentrated sulfuric acid using test tubes, we used a Wifug centrifuge (Wifug Ltd., Bradford, England).

### Analysis.

The following PCB congeners were used as analytical standards: 2,3,3′,4,4′-pentachlorobiphenyl (CB105), 2,2′,3,4,4′,5′-hexachlorobiphenyl (CB138), 2,2′,3,4′,5,5′-hexachlorobiphenyl (CB146), 2,2′,3,3′,4,4′,5-heptachlorobiphenyl (CB170), 2,2′,3,4,4′, 5,5′-heptachlorobiphenyl (CB180), and 2,2′,3,4′,5,5′,6-heptachlorobiphenyl (CB187), and they were purchased from Promochem AB (Ulricehamn, Sweden). 2,2′,4,4′,5,5′-Hexachlorobiphenyl (CB153), 2,3,3′,4,4′,5-hexachlorobiphenyl (CB156), and 2,2′,3,4,4′,5′,6-heptachlorobiphenyl (CB183) were synthesized in house (Bergman et al. 1990; Sundström et al. 1973); 2,3′, 4,4′,5-pentachlorobiphenyl (CB118) and 2,3,3′,4,4′, 5,5′-heptachlorobiphenyl (CB189, internal standard) were synthesized as described elsewhere (Sundström et al. 1973). A larger number of PCB congeners are given here than are given in “Results.” However, for this study, the ones mentioned here were all analyzed. The PCB numbering system as suggested by Ballschmiter et al. (1993) is applied in the present study.

The parent PCB compounds and their OH-PCB metabolites are given in Table 1. The following methoxylated (MeO-PCB) congeners were used as authentic reference standards to quantify the methyl ether derivatives of the hydroxylated PCBs: 4-methoxy-2,3,3′,4′, 5-pentachlorobiphenyl (4-MeO-CB107), 3-methoxy-2,2′,3′,4,4′,5-hexachlorobiphenyl (3′-MeO-CB138), 4-methoxy-2,2′,3,4′,5, 5′-hexachlorobiphenyl (4-MeO-CB146), 3-methoxy-2,2′,4,4′,5,5′-hexachlorobiphenyl (3-MeO-CB153), 4-MeO-2,2′,3,3′,4′,5,5′-heptachlorobiphenyl (4′-OH-CB172), and 4-methoxy-2,2′,3,4′,5,5′,6-heptachloro-biphenyl (4-MeO-CB187). 4-Methoxy-2,3,3′,4′,5,5′,6-heptachlorobiphenyl (4-MeO-CB193) and 4-hydroxy-2,3,3′,4′, 5,5′,6-heptachlorobiphenyl (4-OH-CB193) were synthesized in house (Bergman et al. 1995) and applied as internal standards. The MeO-PCB and OH-PCB congeners are numbered according to Letcher et al. (2000). For this study the following OH-PCBs were measured: OH-CB107, OH-CB153, OH-CB146, OH-CB138, OH-CB187, and OH-CB172 (all presented in “Results”).

### Extraction and cleanup.

The extraction procedure applied in this study is identical to the method described by Hovander et al. (2000). The cleanup procedure that was required to obtain samples pure enough for analysis was a combination of sulfuric acid treatment and silica gel/sulfuric acid column chromatography separations. Both methods are described by Hovander et al. (2000). Before extraction, the samples were spiked with CB189 (2 ng/sample) and 4-OH-CB193 (1 ng/sample). Sample volumes smaller than approximately 4 g of plasma were adjusted to 5 g with an aqueous 1% potassium chloride solution before extraction. For each 10 samples, a solvent blank was run.

The procedure applied for cleanup may be summarized as follows: To approximately 5 g plasma spiked with internal standards, 1 mL 6 M hydrochloric acid was added and mixed well. Thereafter, 2-propanol (6 mL) was added and mixed well; each sample was extracted and reextracted with 6 and 3 mL of hexane:MTBE (1:1), respectively. The phases (a water phase and an organic phase) were separated by centrifugation. The organic phase also underwent a washing step with 4 mL aqueous 1% potassium chloride before reduction of the organic solvent. The lipid residue was determined gravimetrically.

The phenolic compounds were isolated after the extraction from the plasma by using potassium hydroxide (0.5 M in 50% ethanol) and were derivatized to their corresponding methyl ethers by addition of ethereal diazomethane (0.5 mL, 3 hr at 4–8°C) before cleanup and analysis (Hovander et al. 2000).

## Results

In total, 214 pregnant women expressed interest in participating in this study; 104 of them actually participated in the study. Of these 104, a random sample of 51 mother–infant pairs were included in the study.

Clinical characteristics of the mothers and infants are given in Table 2. The mean ± SD maternal age was 31 ± 4 years; mean body mass index (BMI) was 20 ± 3. Infants were born after a gestational age of 40 ± 1 weeks with a birth weight of 3,714 ± 461 g. Fifty-five percent of the infants were male.

The results of all PCB and OH-PCB measurements are presented in Table 3 [mean (range)]. The sum PCB (sum of six congeners) in maternal plasma was 268 (113–619) ng/g lipid [mean (range)], compared with 345 (78–809) ng/g lipid in cord blood. The sum OH-PCB (sum of six congeners) in maternal blood was 54 (14–125) ng/g lipid weight compared with 114 (49–244) ng/g lipid in cord blood. Expressed per gram fresh weight—a more suitable way of expressing OH-PCB values because they are more hydrophilic than are PCBs—the levels were 0.340 (nondetectable to 0.622) ng/g fresh weight in maternal blood compared with 0.180 (nondetectable to 0.407) ng/g fresh weight in cord blood. The lipid content of maternal plasma was considerably higher than that of the fetus, 0.7 versus 0.2 g/100 mg plasma.

**Figure 1:**
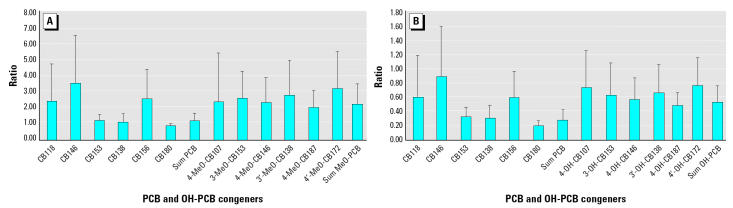
Ratio cord versus maternal plasma expressed (*A*) per lipid weight and (*B*) per fresh weight (mean ± SD).

The ratios of cord versus maternal plasma concentrations for PCB and OH-PCB congeners, expressed in nanograms per gram lipid as well as nanograms per gram plasma, are shown in Figure 1. The sum PCBs have a ratio ± SD of 1.3 ± 0.56 when expressed per gram of lipid. The sum OH-PCBs have a ratio of 2.2 ± 1.33 when expressed per gram of lipid and a ratio of 0.5 ± 0.23 when expressed per gram of plasma.

**Figure 2:**
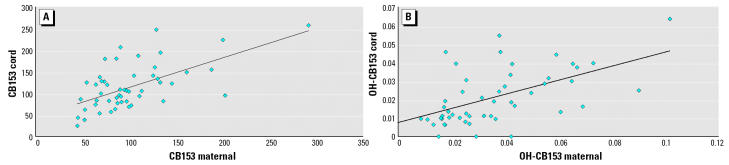
Correlation between maternal and cord plasma levels for (*A*) PCB-153 (expressed per lipid weight) and (*B*) OH-PCB-153 (expressed per fresh weight).

Table 4 shows the correlation between the maternal and cord levels for both the PCBs and the OH-PCBs. Except for CB118 and CB156, there was a significant correlation between the maternal and cord plasma levels. This indicates a transfer of these compounds across the placental barrier. Figure 2 shows the correlation between PCB and OH-PCB levels between maternal and cord plasma.

The correlations between the parent compound and the resulting OH-PCB for both maternal plasma and cord plasma are given in Table 5. There was a significant correlation between the parent compound and resulting OH metabolite for all congeners.

## Discussion

In this study, we found that OH-PCBs, metabolites of PCBs, are detectable in plasma of pregnant women who are exposed to background levels of PCBs in the Netherlands. Both the PCB pollutants and their OH-PCB metabolites are also detectable in cord plasma. Second, plasma levels of OH-PCBs in umbilical cord plasma are 50% of the levels in the mothers, indicating a considerable placental transfer. The placental transfer of OH-PCBs may be explained from their strong binding to transthyretin (TTR) and active transport across the placenta. PCBs, in contrast, are neutral lipophilic compounds strongly distributed to lipids and therefore less readily cross the placenta.

The low ratio in PCBs when expressed per gram of plasma may be explained by the fact that a newborn infant consists of 15% fat, in contrast to 25% fat in adults. In our study, the lipid content of cord plasma is about 0.2% lipids compared with 0.7% lipids in the mother. The PCB body burden of the infant expressed per gram of body weight therefore is lower than the body burden of the mother, although the PCB levels are equal or slightly higher when expressed per gram of lipid.

Because of the different transfer mechanism of OH-PCBs, the infant will have a body burden, expressed per body weight of OH-PCBs, of 50–70% of their mothers, an intriguing observation making OH-PCBs possibly more important from a risk assessment viewpoint than initially thought.

Our finding that there is a correlation between the maternal and cord levels of both PCBs and OH-PCBs further supports the transplacental transfer of both compounds.

PCBs can reach the fetus only by transplacental transfer. OH-PCBs in the fetus can be the result of transplacental transfer as well as hydroxylation by the fetus itself. From our observational study, no firm conclusions can be drawn regarding the source of OH-PCBs in the fetus. Levels of OH-PCBs were, on average, approximately 50% of maternal levels. At the same time, the correlation between parent PCB and resulting OH-PCB was stronger in the fetus than in the pregnant mother. The fetus excretes OH-PCBs to the mother, whereas the mother excretes OH-PCBs in feces and/or urine. That the correlation between the parent compound and the OH congener is rather weak in the mother can be explained by differences in kinetics between the PCBs and OH-PCBs. PCBs have a half-life longer than that of OH-PCBs.

Levels of PCBs found in this study are almost equal to levels we have found in the Netherlands in a comparable group of healthy pregnant women (Koopman-Esseboom et al. 1994b, 1994c). Plasma levels found in this study versus levels found 10 years ago are, for CB118, 0.19 versus 0.16 ng/g plasma; for CB138, 0.50 versus 0.60 ng/g; for CB153, 0.70 versus 0.91 ng/g; and for CB180, 0.30 versus 0.54 ng/g. Although the samples are analyzed in different laboratories by different methods, these results might indicate that PCB levels in the Netherlands do seem to have hardly declined in these 10 years. We cannot compare the OH-PCB levels because OH-PCB levels have not been measured before in the Dutch population.

One limitation to this study is the time difference between the blood taken from the pregnant mother and cord blood. We do not believe, however, that this time difference influenced our results. PCB levels in the mother are the result of lifelong exposure and do not change during pregnancy (Koopman-Esseboom et al. 1994a). Most likely, the conversion of PCB to OH-PCB does not change during pregnancy. Although we did not measure the level of OH-PCB at more times during pregnancy, we expected the levels of both PCBs and OH-PCBs to be constant during pregnancy and therefore accepted the time difference between maternal samples and cord samples, caused by practical considerations.

The mother–infant pairs included in this study were a random selection from a larger cohort of mother–infant pairs. The total group was included on a voluntary basis in our study. Although we expected to have a random sample of mothers in our region, we cannot exclude some bias in the mothers who volunteered to enter the study. Mothers might have had some concerns regarding these environmental compounds. These could be mothers who select their food carefully, but also mothers without such opportunities for food selection who are therefore concerned. Altogether we believe, however, that our population is a valid representation of the population in our region.

Sjödin et al. (2000) measured PCB and OH-PCB levels in Swedish and Latvian fishermen consuming either low or high amounts of fish from the Baltic Sea, known to be highly polluted by contaminants. They found CB153 levels in Swedish fishermen ranging from 226 ng/g lipid in low fish consumers to 534 ng/g lipid in high fish consumers. The CB153 levels in the pregnant women in our study were 101 ng/g lipid (range, 43–293). Sjödin et al. (2000) also measured OH-PCB levels in their subjects. The 4-OH-CB187 levels ranged from 31 ng/g lipid in low fish consumers to 176 ng/g lipid in high fish consumers from Latvia and from 43 to 75 ng/g lipid in their Swedish counterparts. In the pregnant women in our study, we found much lower 4-OH-CB187 levels of 20 ng/g lipid (range, 6.6–49).

Recently, Sandau et al. (2002) also analyzed OH-PCBs in umbilical cord plasma of neonates from coastal populations in Québec. They measured OH-PCB levels in three different areas. In one area (southern Québec), individuals were exposed to background levels of PCBs, and in the other two (Nunavik and Lower North Shore) they were selected because of their high fish consumption. The OH-PCB concentration range for OH-CB187 was 10–250 pg/g plasma; OH-CB146, 4–507 pg/g plasma; OH-CB153, 3–74 pg/g plasma; OH-CB107, 3–168 pg/g plasma; OH-CB138, 3–92 pg/g plasma; and OH-CB172, 1–75 pg/g plasma (Sandau et al. 2002). The concentrations measured in Québec are higher than those found in our study. This can be explained by the level of contamination and the mainly fish diet of Québec. Also remarkable is a different pattern in OH-PCBs between the study in Québec and our study. This might indicate a different source of PCBs.

OH-PCBs are considered endocrine disruptors in animals, with effects on thyroid hormones, estrogens, and testosterone. Animal studies have shown a significant reduction of thyroid hormones in the brain after exposure to OH-PCBs. The reduction in thyroid hormones is related to the binding of OH-PCBs to TTR, which can be explained by the strong structural resemblance between OH-PCB and thyroxine (Brouwer et al. 1998; Ghosh et al. 2000). Some OH-PCBs have > 60% higher affinity for TTR than does thyroxine itself (Ghosh et al. 2000; Meerts et al. 2000, 2002). In humans, thyroid hormones are mainly bound to thyroxine-binding globulin (TBG) (Brouwer et al. 1998). Whether the binding of OH-PCBs to TTR has negative effects in humans is unknown.

Meerts (2001) observed a significant prolongation of estrous cycles in the offspring of pregnant rats exposed to OH-CB107 but found no effect on their reproductive performance. Also, Meerts (2001) observed an impaired habituation in male rat offspring but not in female offspring.

Whether OH-PCBs do have an effect in the human on either the thyroid or sex hormones is unknown. TBG and not TTR is the main transport protein of thyroxine in humans. TTR, however, might be important for the transfer of thyroid hormones into the brain. Further studies in humans are needed to elucidate if OH-PCBs have a negative effect on the human fetus or older individuals.

## Conclusion

Our study indicates that OH-PCBs can be found in the plasma of healthy pregnant women in the Netherlands. The level of OH-PCBs in cord plasma is approximately 50% of levels found in the mother. So both PCBs and OH-PCBs cross the placenta. In both cord and maternal plasma, OH-PCBs are correlated with the respective parent PCBs. The only way to reduce the exposure of the fetus to these potentially toxic compounds is to reduce the body burden of PCBs in pregnant women.

## Figures and Tables

**Table 1:** PCB congeners and their respective OH-PCB metabolites.

Parent PCB	Metabolite
CB105	4-OH-CB107
CB118	4-OH-CB107
CB138	4-OH-CB146
CB138	3′-OH-CB138
CB153	4-OH-CB146
CB153	3-OH-CB153
CB170	4′-OH-CB172
CB180	4′-OH-CB172
CB187	4-OH-CB187

**Table 2:** Characteristics of the study group (*n* = 51).

Characteristic	Value
Maternal age (years; mean ± SD)	31 ± 4
Weight gain during pregnancy (kg; mean ± SD)	13.3 ± 6
Mother’s BMI (mean ± SD)	20 ± 3
Parity, first-born/second- or third-born (%)	33/67
Smoking during pregnancy, yes/no (%)	21/79
Alcohol during pregnancy, yes/no (%)	23/77
Sex of child, male/female (%)	55/45
Gestational age (weeks; mean ± SD)	40 ± 1
Apgar score 1 min [median (range)]	9 (4–10)
Birth weight (g; mean ± SD)	3,714 ± 461

**Table 3:** PCB and OH-PCB concentrations in corresponding maternal and cord plasma samples [mean (range); *n* = 51].

PCB	Maternal PCB		Cord PCB			Maternal OH-PCB		Cord OH-PCB	
congener	lw (ng/g)	fw (ng/g)	lw (ng/g)	fw (ng/g)	OH-PCB congener	lw (ng/g)	fw (ng/g)	lw (ng/g)	fw (ng/g)
CB118	28 (8–69)	0.188 (0.054–0.452)	56 (9–277)	0.093 (0.013–0.470)	4-OH-CB107	10 (0.8–38)	0.060 (ND–0.183)	14 (4–354)	0.022 (ND–0.048)
CB146	10 (2–29)	0.069 (0.015–0.312)	30 (1–106)	0.050 (0.002–0.154)	3-OH-CB153	5 (1.4–13)	0.035 (ND–0.101)	14 (3–38)	0.021 (ND–0.063)
CB153	101 (43–293)	0.700 (0.248–3.514)	115 (25–252)	0.193 (0.037–0.412)	4-OH-CB146	10 (3–27)	0.063 (ND–0.129)	23 (8–58)	0.036 (ND–0.097)
CB138	73 (32–171)	0.496 (0.183–2.040)	77 (20–213)	0.130 (0.029–0.399)	3′-OH-CB138	7 (1.3–26)	0.045 (ND–0.166)	18 (6–50)	0.028 (ND–0.079)
CB156	12 (4–22)	0.084 (0.028–0.215)	29 (7–96)	0.047 (0.013–0.135)	4-OH-CB187	20 (7–49)	0.022 (ND–0.048)	38 (17–69)	0.061 (ND–0.115)
CB180	44 (15–93)	0.300 (0.090–0.961)	37 (11–88)	0.063 (0.019–0.144)	4′-OH-CB172	2 (0.4–6)	0.015 (ND–0.034)	7 (2–18)	0.011 (ND–0.030)
∑PCB	268 (113–619)	1.837 (0.645–7.432)	345 (78–809)	0.585 (0.134–1.370)	∑OH-PCB	54 (14–125)	0.340 (ND–0.622)	114 (49–244)	0.180 (ND–0.407)
Lipid (%)	0.7 (0.4–1.2)		0.2 (0.1–0.3)		Lipid (%)	0.7 (0.4–1.2)		0.2 (0.1–0.3)	

Abbreviations: fw, fresh weight; lw, lipid weight; ND, nondetectable.

**Table 4:** Correlation between maternal and cord plasma levels of PCBs.

PCB congener (g lipid)	Correlation coefficient	OH-PCB congener (g fresh weight)	Correlation coefficient
CB118	0.03	4-OH-CB107	0.15
CB146	0.34	3-OH-CB153	0.54
CB153	0.57	4-OH-CB146	0.54
CB138	0.36	3′-OH-CB138	0.57
CB156	0.15	4-OH-CB187	0.55
CB180	0.79	4′-OH-CB172	0.58
Sum	0.43	Sum	0.52

**Table 5:** Correlation between parent compound and hydroxy metabolite in maternal and cord plasma.

Parent PCB congener (g lipid)	OH-PCB metabolite congener (g fresh weight)	Correlation coefficient in maternal sample	Correlation coefficient in cord sample
CB118	4-OH-CB107	0.21	0.46
CB138	4-OH-CB146	0.38	0.58
CB138	3′-OH-CB138	0.26	0.33
CB153	4-OH-CB146	0.39	0.49
CB153	3-OH-CB153	0.27	0.33
CB180	4′-OH-CB172	0.48	0.45
